# Diethylcarbamazine Reduces Chronic Inflammation and Fibrosis in Carbon Tetrachloride- (CCl_**4**_-) Induced Liver Injury in Mice

**DOI:** 10.1155/2014/696383

**Published:** 2014-10-13

**Authors:** Sura Wanessa Santos Rocha, Maria Eduarda Rocha de França, Gabriel Barros Rodrigues, Karla Patrícia Sousa Barbosa, Ana Karolina Santana Nunes, André Filipe Pastor, Anne Gabrielle Vasconcelos Oliveira, Wilma Helena Oliveira, Rayana Leal Almeida Luna, Christina Alves Peixoto

**Affiliations:** ^1^Laboratory of Ultrastructure, Aggeu Magalhães Research Center (CPqAM), Avenida Professor Moraes Rego, s/n, Cidade Universitária, 50740-465 Recife, PE, Brazil; ^2^Department of Entomology, Aggeu Magalhães Research Center (CPqAM), Avenida Professor Moraes Rego, s/n, Cidade Universitária, 50740-465 Recife, PE, Brazil; ^3^Laboratory of Virology and Experimental Therapy, Aggeu Magalhães Research Center (CPqAM), Avenida Professor Moraes Rego, s/n, Cidade Universitária, 50740-465 Recife, PE, Brazil; ^4^Laboratory of Microscopy and Microanalisis, Northeastern Center for Strategic Technologies (CETENE), Avenida Professor Luiz Freire, Cidade Universitária, 50740-540 Recife, PE, Brazil

## Abstract

This study investigated the anti-inflammatory effects of DEC on the CCl_4_-induced hepatotoxicity in C57BL/6 mice. Chronic inflammation was induced by i.p. administration of CCl_4_ 0.5 *μ*L/g of body weight through two injections a week for 6 weeks. DEC (50 mg/kg) was administered by gavage for 12 days before finishing the CCl_4_ induction. Histological analyses of the DEC-treated group exhibited reduced inflammatory process and prevented liver necrosis and fibrosis. Immunohistochemical and immunofluorescence analyses of the DEC-treated group showed reduced COX-2, IL1*β*, MDA, TGF-*β*, and *α*SMA immunopositivity, besides exhibiting decreased IL1*β*, COX-2, NF*κ*B, IFN*γ*, and TGF*β* expressions in the western blot analysis. The DEC group enhanced significantly the IL-10 expression. The reduction of hepatic injury in the DEC-treated group was confirmed by the COX-2 and iNOS mRNA expression levels. Based on the results of the present study, DEC can be used as a potential anti-inflammatory drug for chronic hepatic inflammation.

## 1. Introduction

Chronic liver disease leads to several complications, with high morbidity and mortality rates. A worldwide health problem, chronic liver inflammation is a progressive disease with a slow stage, causing liver dysfunction, characterized by the formation of fibrosis [1]. The chronicity of inflammation, as well as the type of inflammation (i.e., Th2 versus Th1), is often important in many types of liver disease, as is the interplay between inflammation and environmental/metabolic/genetic factors [2]. Excessive consumption of alcohol and viral hepatitis are among the most common causes of liver disease [3]. The liver is the main organ responsible for the metabolism of drugs and toxic chemicals and, consequently, it is the primary target organ for nearly all toxic chemicals [4].

Carbon tetrachloride (CCl_4_) is a well-known hepatotoxin that is widely used to induce toxic hepatic injuries in experimental animals models [5, 6]. Hepatotoxicity is believed to result from two events: the first involves CCl_4_ metabolism by cytochrome P-450 to the trichloromethyl radical (CCl_3_^•^), part of which generates the trichloromethyl peroxyl radical (OOCCl_3_^•^) [7, 8], which leads to lipid peroxidation [9]; the second involves the activation of Kupffer cells, which is accompanied by the production of proinflammatory mediators [10]. Carbon tetrachloride (CCl_4_) should be an effective stimulus for prostaglandin synthesis in hepatocytes, since it is believed to release arachidonic acid in the liver through the activation of phospholipase A2 [11, 12].

Progression of the disease involves various proinflammatory molecules such as interleukins, cytokines, and nuclear factor-*κ*B (NF-*κ*B) [13–15]. The activated NF-*κ*B, if translocated into the nucleus, will facilitate the transcription of many downstream genes, including inducible nitric oxide synthase (iNOS) and cyclooxygenase-2 (COX-2), which are key mediators in the recruitment of inflammatory cells [16, 17]. The iNOS-derived nitric oxide production, activated downstream of NF-*κ*B, is followed by the generation of reactive oxygen species and other free radicals that are detrimental to cells. Cellular lipids are easily attacked by free radicals, resulting in an intracellular accumulation of malondialdehyde (MDA) [18]. Although the relationships between oxidative stress, cytotoxic cytokines, and liver cell injuries are not fully understood, NF-*κ*B is considered to play an important role in liver cell injuries. Studies have shown an increased activation of NF-*κ*B through oxidative stress induced by carbon tetrachloride in liver injuries [19, 20].

After a chronic liver injury of any etiology, the damaged hepatocytes, their membrane components, metabolites of toxic agents, and infiltrating inflammatory cells activate Kupffer cells, releasing soluble agents, including cytokines, such as platelet-derived growth factor (PDGF), transforming growth factor-*β*1 (TGF-*β*), tumor necrosis factor-a (TNF-*α*), and endothelin-1 (ET-1), as well as reactive oxygen species (ROS) [21]. These factors act on the hepatic stellate cells (HSCs), which undergo a response known as activation and start expressing new receptors, such as PDGF and TGF-*β* receptors, and new proteins, such as a-smooth muscle actin (*α*-SMA). The activation of the HSCs triggers the expression of *α*-SMA and increases cellular proliferation and extracellular matrix accumulation [22]. Activated HSCs are considered to play a role in the excessive deposition of ECM proteins, such as type I collagen (Col-1), and in the disruption of normal parenchyma during the hepatic fibrogenesis process. Chronic inflammation and the repeated sustenance of liver injuries, such as those associated with hepatitis and alcoholic liver disease, usually progress to liver cirrhosis and hepatocarcinogenesis [23].

The inhibition of proinflammatory cytokines, enzymes, transcription nuclear factor, and free radicals could offer a new therapeutic strategy against inflammatory liver disease.

Diethylcarbamazine (DEC) has anti-inflammatory properties as a result of its interference with the arachidonic acid metabolism, which involves lipoxygenase (LOX) and cyclooxygenase (COX) enzymes [24–27]. DEC led to reductions in lung injury, PMNs migration, the formation of NO production, and the release of proinflammatory cytokines and COX-2 in a model of acute inflammation induced by carrageenan [28]. DEC is a potential drug for the treatment of acute lung inflammation, for Queto et al. demonstrated that DEC plays an important role in blocking pulmonary eosinophilic inflammation in mice sensitized with ovalbumin, effectively preventing the effects of subsequent airway resistance, Th1/Th2 cytokine production, pulmonary eosinophil accumulation, and eosinophilopoiesis* in vivo *and* ex vivo *[29]. DEC effects have contributed to the reduction of hepatic abnormalities caused by malnutrition [30]. In addition, DEC has decreased liver injuries, in an experimental alcoholism model, and revealed a clinical potential for therapeutic anti-inflammatory applications [15].

The aim of the present study was to investigate the protective effects of DEC against chronic CCl_4_-induced damage and a possible mechanism for its anti-inflammatory and hepatoprotective activity.

## 2. Materials and Methods

### 2.1. Animals

Forty male 5-week-old C57BL/6 mice, weighting 15-16 g, were used in all experiments. The mice were examined to determine their health status and acclimated to the laboratory environment of 23-24°C. They were kept in a 12/12 h day/night cycle photoperiod. The animals were housed in metal cages and fed a standard diet with water* ad libitum*. The animal studies Ethics Committee of the Oswaldo Cruz Institute approved all the experiments reported herein under protocol number 11/2010.

### 2.2. Diethylcarbamazine Solutions

The solutions were composed of distilled water and DEC (Farmanguinhos, FIOCRUZ, Brazil) and were adjusted in accordance with the body weight of each animal. They were administered by gavage (0.2 mL). The control group (C) only received distilled water via the same administration route [15, 30, 31].

### 2.3. Experimental Design

Chronic inflammation was induced by i.p. administration of CCl_4_ 0.5 *μ*L/g of body weight (Sigma-Aldrich, St. Louis, MO, USA) dissolved in olive oil (final volume, 0.1 mL per mouse) [32]. There were two injections a week for 6 weeks. DEC (50 mg/kg) was administered by gavage for 12 days before finishing the liver injury. The control groups received distilled water via the same route. The C57BL/6 mice were separated into four groups (*n* = 10): (1) the control group (C), which received just distilled water; (2) the DEC-treated group (DEC); (3) the CCl_4_ group (CCl_4_); and (4) the CCl_4_ plus DEC group (CCl_4_ + DEC).

### 2.4. Histological Analysis

Liver fragments were fixed in 10% formalin for 24 hours, before being processed and embedded in paraffin [30]. Five sections of 4-5 *μ*m from each group were cut and mounted on glass slides. The slices were stained with hematoxylin-eosin and examined by an inverted microscope (Observer Z1, Zeiss MicroImaging, GmbH) equipped with a camera and 4.7.4 image analysis software (AxionCam MRm Zeiss) at a magnification of 400x. The fibrosis areas were quantified in five random fields on each slide using GIMP 2.6 imaging software [15].

### 2.5. Measurement of Hepatic Collagen Content

The hepatic collagen content was also assessed by the Sirius-red staining of five paraffin-embedded sections. Sirius-red positive areas were analyzed in five random fields (magnification ×400) on each slide and quantified using GIMP 2.6 imaging software [15].

### 2.6. Electron Transmission Microscopy Assays

The fragments of liver were fixed in a solution containing 2.5% glutaraldehyde and 4% formaldehyde in 0.1 M cacodylate buffer. After fixation, the samples were washed twice in the same buffer and then postfixed in a solution containing 1% osmium tetroxide, 2 mM calcium chloride, and 0.8% potassium ferricyanide in 0.1 M cacodylate buffer with a pH of 7.2, dehydrated in acetone and embedded in Epon 812 resin (Sigma Company, St. Louis, MO). Polymerization was carried out at 60°C for 2 days. Ultrathin sections were collected on 300-mesh copper grids, counterstained with uranyl acetate and lead citrate, and examined with a Morgani FEI transmission electron microscope [15].

### 2.7. Immunohistochemistry (IHC)

Five sections (5 *μ*m in thickness) from each group were cut and adhered to slides treated with 3-amino-propyl-triethoxy-silane (APES (Sigma, USA)). The sections were deparaffinized with xylene and rehydrated in graded ethanol (100 to 70%). To increase epitope exposure, the sections were heated for 30 minutes in a sodium citrate buffer (0.01 M, pH 6.0). To minimize endogenous peroxidase activity, the slides were treated with 0.3% (v/v) H_2_O_2_ in water for five minutes. The sections were washed with 0.01 M PBS (pH 7.2) and then blocked with 1% BSA and 0.2% Tween 20 in PBS for 1 h at room temperature. The sections were then incubated for 12 hours at 4°C with a monoclonal antibody anti-cyclooxygenase (COX-2, Abcam, ab15191), an antibody against transforming growth factor-beta (TGF-*β*, Abcam, ab66043), and anti-mouse interleukin 1 (IL-1*β*, Abcam, ab9722). The optimal concentration used was 1 : 100 for these antibodies. The antigen-antibody reaction was visualized with avidin-biotin peroxidase (Dako Universal LSAB + Kit, Peroxidase), using 3.3-diaminobenzidine as the chromogen. The slides were counterstained in hematoxylin. Positive staining resulted in a brown reaction product. Negative controls were treated as above, with the exception of the first antibody, which was omitted. Five pictures at the same magnification were quantitatively analyzed using GIMP 2.6 software (GNU Image Manipulation Program, UNIX platforms).

### 2.8. Immunofluorescence (IF)

After anesthesia, the animals were euthanized and fragments of liver were fixed in a solution of 4% paraformaldehyde (Sigma-Aldrich) (40 mL) in 0.1 M phosphate (sodium phosphate monobasic and dibasic heptahydrate, Sigma-Aldrich) buffered saline (PBS), with a pH of 7.2 for two hours. The livers were immersed in 15% sucrose overnight, followed by 30% sucrose for a second night (36 hours total). The specimens were then embedded in OCT-Tissue Tek compound (Sakura Finetek, Torrance, CA, USA), frozen in n-hexane (Dinâmica, São Paulo, SP, Brazil) and cooled with liquid nitrogen. Cryosections (8 *μ*m thick) were permeabilized (0.3% Triton X-100) and incubated for 1 h with blocking solution (3% BSA plus 0.2% Tween 20 in Tris buffered saline). Subsequently, the sections were incubated with antibodies for COX-2 (Abcam, ab15191), *α*SMA (Abcam, ab66043), and IL-1*β* (Abcam, ab9722) (both 1 : 100). The sections were incubated with primary antibodies overnight and then incubated with polyclonal Cy3-conjugated secondary antibodies (Jackson, cat. no. 705-165-147) against rabbit immunoglobulin (1 : 200) for 1 h. The slides were washed and mounted in fluorescent Prolong Gold Antifade medium (Life Technologies, cat. no. P36930) for observation under an inverted fluorescence microscope (Zeiss MicroImaging GmbH), which was equipped with a camera (Zeiss AxioCam MRM) and Release 4.7.2 image analysis software.

### 2.9. Western Blot

The livers were quickly dissected and then homogenized in a Wheaton Overhead Stirrer (no. 903475) using the following extraction cocktail: 10 mM ethylenediamine tetraacetic acid (EDTA); 2 mM phenylmethylsulfonyl fluoride (PMSF); 100 mM sodium fluoride; 10 mM sodium pyrophosphate; 10 mM sodium orthovanadate (NaVO_4_); 10 mg aprotinin and 100 mM Tris (hydroxymethyl)aminomethane (pH 7.4). Homogenates were centrifuged at 3000 ×g for 10 min and the supernatant was collected and stored at −70°C until its use in the immunoblotting. Protein levels were determined using the Bradford method, with bovine serum albumin as the standard [33]. The proteins (40 mg) were separated with 10% (IL10, IL-1*β*, NF-*κ*B-p65, COX-2, IFN*γ*, and TGF-*β*) sodium dodecyl sulfate-polyacrylamide by gel electrophoresis under reduced conditions and were electrophoretically transferred onto nitrocellulose membranes (Bio Rad, CA, USA, Ref. 162-0115). After blocking overnight at 4°C with 5% nonfat milk in TBS-T (Tris buffered saline 0.1% plus 0.05% Tween 20, pH 7.4), the membranes were incubated at room temperature, for 3 h, with rabbit polyclonal antibodies anti-IL10 and anti-IL-1*β* (both 1 : 1000 dilution; Abcam, USA), anti-IFN*γ*, and anti-TGF-*β* (both 1 : 2000 dilution, Abcam, CA, USA) and with rabbit polyclonal antibodies anti-NF-*κ*B-p65 and anti-COX-2 (both 1 : 1500 dilution, Abcam, CA, USA), diluted in buffer solution TBS-T containing 3% nonfat milk. After washing (six times, 10 min each) in TBS-T, the membranes were further reacted with horseradish peroxidase-conjugated anti-rabbit secondary antibody (1 : 8000 (Ref. A6154) dilution, Sigma, USA), diluted in TBS-T with 1% nonfat milk, for 1 h 30 min, at room temperature. An enhanced chemiluminescence reagent (Super Signal, Pierce, Ref. 34080) was used to visualize the labeled protein bands and the blots were developed on X-ray film (Fuji Medical, Kodak, Ref. Z358487-50EA). For quantification, the density of pixels of each band was determined by the Image J 1.38 program (available at http://rsbweb.nih.gov/ij/download.html; developed by Wayne Rasband, NIH, Bethesda, MD). The results were confirmed in three sets of experiments for each protein investigated. The immunoblot for *β*-actin was used as a control for the above protein blots. After protein blot visualization with enhanced chemiluminescence, the protein antibodies were stripped from the membranes, which were reprobed with monoclonal anti-*β*-actin antibody (1 : 1000 dilution, Sigma, USA). Protein densitometry was subsequently carried out.

### 2.10. RNA Isolation, RT-PCR, and Real-Time Quantitative PCR

Total RNA from mouse tissues was isolated using Trizol reagent (Invitrogen, Carlsbad, CA, USA). The RNA was treated with RNase-free DNase I and amplified with oligo (dT) primer, using the SuperScript First-Strand Synthesis System for RTPCR (Invitrogen). Then, 1 *μ*g of total RNA was reverse-transcribed using the QuantiTec Reverse Transcription kit (Qiagen, Hilden, Germany), using random hexameric primers, according to the manufacturer's instructions. Quantitative real-time PCR was performed with the SYBR Green PCR system (Applied Biosystems, Foster City, CA, USA), using GAPDH as an internal control for normalization. RTqPCR was carried out with an ABI PRISM 7500 instrument (Applied Biosystems, CA, USA). The forward and reverse primers used for each gene were as follows: 5′-TGAGCAACTATTCCAAACCAGC-3′ and 5′-GCACGTAGTCTTCGATCACTATC-3′ for COX-2; 5′-GTTCTCAGCCCAACAATACAAGA-3′ and 5′-GTGGACGGGTCGATGTCAC-3′ for iNOS; and 5′-AGGTCGGTGTGAACGGATTTG-3′ and 5′-TGTAGACCATGTAGTTGAGGTCA-3′ for GAPDH (endogenous control). All reactions were performed in triplicate and included the following: 1 *μ*L of cDNA; 5 *μ*M of each primer; 2x SYBR Green PCR Master Mix (Applied Biosystems); and water added to a final volume of 25 *µ*L. The relative amount of mRNA was determined using the comparative threshold (Ct) method by normalizing target cDNA Ct values to that of GAPDH. Fold increase ratios were calculated relative to the control (basal conditions) for each group using the formula 2*e* − ΔΔCt.

### 2.11. Determination of Serum Oxide Nitric (NO) Activity

The NO_2_^−^ levels in serum were determined by a method based on the Griess reaction [34]. The Griess colorimetric reaction was used to measure nitric oxide, which involved detecting nitrite (NO_2_^−^) and the oxidation of NO in the plasma. In duplicate, 50 *μ*L of the plasma was added to a 96-well ELISA plate, followed by the same volume of Griess reagent, which is composed of 1% sulfanilamide, diluted in 2.5% H_3_PO_4_ (solution A) and N-1-naphtyl-ethtylenodiamina, also diluted in 2.5% H_3_PO_4_ (solution B). A solution of sodium nitrite in an initial concentration of 100 *μ*M was serially diluted in PBS to prepare the standard curve. After incubation for 10 minutes in the dark, a reading was performed by the spectrophotometer at 490 nm. The absorbance of different samples was compared with the standard curve and the results were expressed as the mean ± standard error of the duplicate, using GraphPad Prism software (v. 5.0).

### 2.12. Statistical Analyses

GraphPad Prism software (version 5) was used for statistical analyses. Data were expressed using mean ± standard deviation. Differences between the control and treated groups were analyzed by analysis of variance (ANOVA), followed by Dunnett's test, Tukey's test, or the *t*-test as post hoc tests. Probability values less than 0.05 were considered significant.

## 3. Results

### 3.1. Histopathology

**Figure 1:**
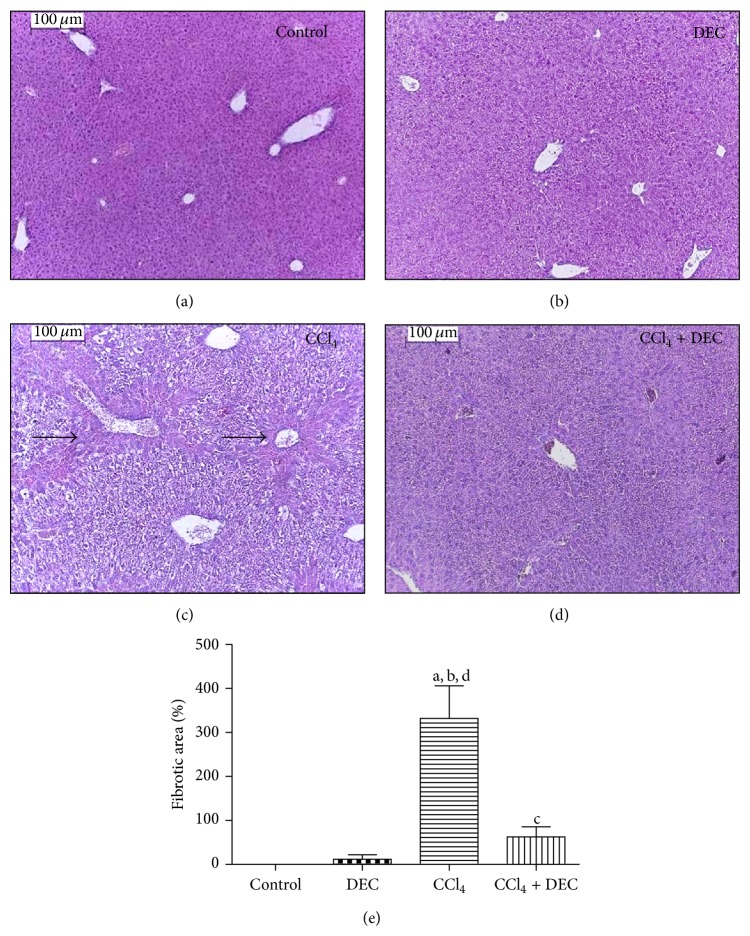
Micrograph of hepatocytes. (a) Control group showing typical morphology, (b) group treated with 50 mg/kg DEC (DEC), (c) CCl_4_ group (CCl_4_), and (d) CCl_4_ plus 50 mg/kg DEC group (CCl_4_ + DEC). Magnification = 100 *μ*m. H&E stain. (e) Quantification fibrosis area (mean ± S.D., *n* = 5). ^a^ *P* < 0.05 when compared with control group; ^b^ *P* < 0.05 when compared with DEC group; ^c^ *P* < 0.01 when compared with CCl_4_ group; ^d^ *P* < 0.05 when compared with CCl_4_ + DEC group. Fibrotic areas (arrows).

Histological analysis of the control group mice (Figure 1(a)) showed normal liver architecture. Animals from the DEC group exhibited similar patterns (Figure 1(b)). In the CCl_4_ group, several histological characteristics of a persistent inflammatory process were found, particularly pericentral necrosis and fibrosis, vacuolar fatty change, mild inflammatory cell infiltration, cytoplasmic degeneration, and nuclear disorganization (Figure 1(c)). DEC treatment of animals exposed to CCl_4_ completely prevented liver necrosis and fibrosis. It also attenuated the inflammatory infiltrates, with minimal hepatic damage (Figure 1(d)).

### 3.2. Collagen Analysis

**Figure 2:**
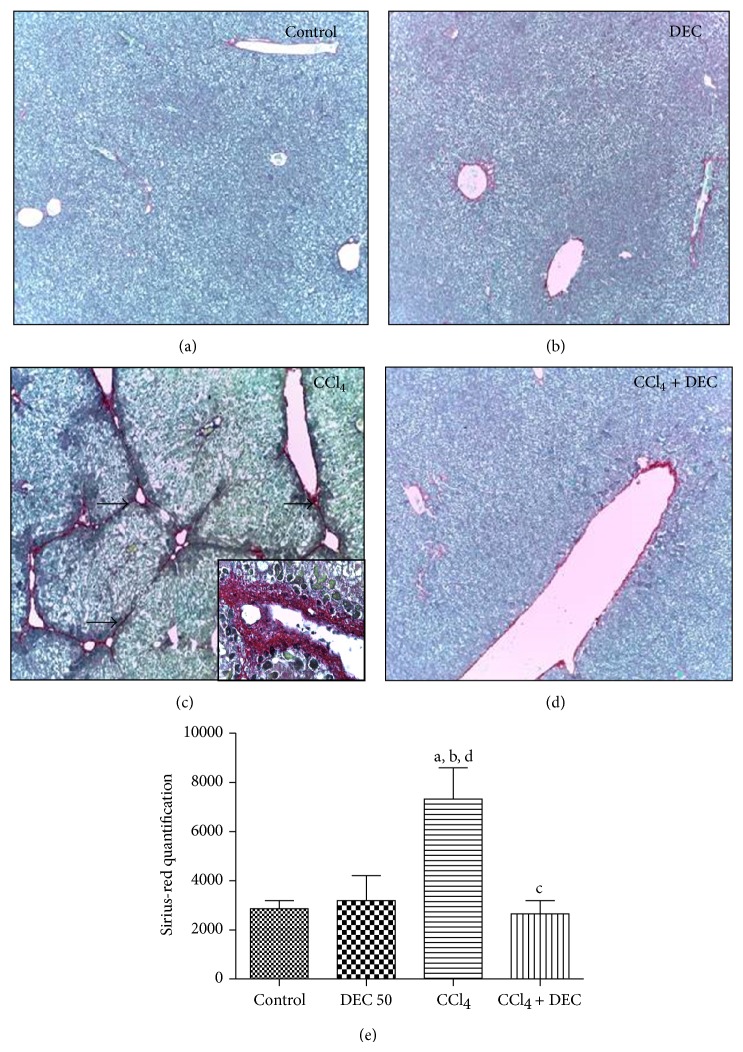
Micrograph of hepatocytes. (a) Control group showing typical morphology, (b) group treated with 50 mg/kg DEC (DEC), (c) CCl_4_ group (CCl_4_), and (d) CCl_4_ plus DEC group (CCl_4_ + DEC). Magnification = 100 *μ*m. Sirius-red staining. (e) Quantification of fibrosis area (mean ± S.D., *n* = 5). ^a^ *P* < 0.05 when compared with control group; ^b^ *P* < 0.05 when compared with DEC group; ^c^ *P* < 0.01 when compared with CCl_4_ group; ^d^ *P* < 0.05 when compared with CCl_4_ + DEC group. Collagen depositions (arrow).

Hepatic fibrosis was assessed by Sirius-red staining (marking type I and type III collagen fibers). The control and DEC groups did not exhibit significant Sirius-red positive areas (Figures 2(a) and 2(b)). The CCl_4_ group exhibited significant collagen deposition around the portal spaces and in fibrotic areas (Figure 2(c)). However, the CCl_4_ + DEC group exhibited a reduction in collagen deposition along the hepatic tissue, as observed in the control group (Figure 2(d)). Positive Sirius-red areas were quantified by GIMP 2.6 imaging software and are illustrated in Figure 2(e).

### 3.3. Ultrastructural Assays

**Figure 3:**
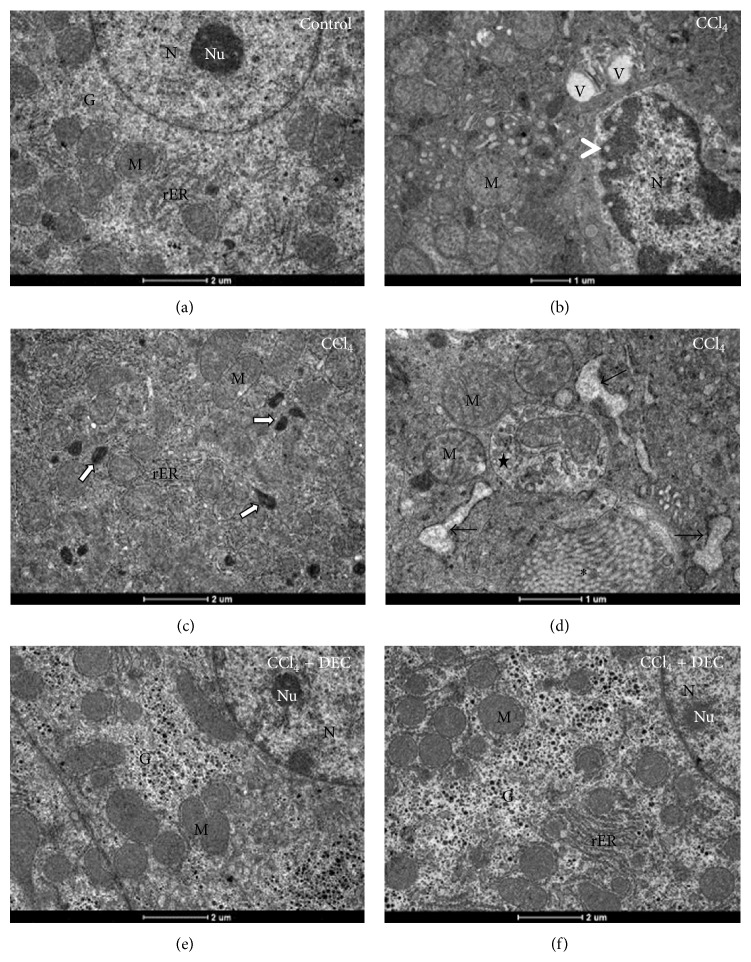
Ultrathin sections of hepatocytes. (a) Control group; (b), (c) and (d) CCl_4_ group; (e) and (f) DEC + CCl_4_ group. Note that the chronic cell injury exhibits swollen mitochondria, several vacuoles (V) and lyses of chromatin, characteristic of a necrosis process (head arrows). Note also the collagen fibers (asterisk), rER dilatation (black arrows), and an autophagosome containing mitochondria (star). Mitochondria (M), glycogen (G), nucleus (N), nucleolus (Nu), rough endoplasmic reticulum (rER), and peroxisomes (white arrows). Bar: 1 and 2 *μ*m.

The ultrastructural analysis of hepatocytes of the control group exhibited typical morphological patterns, such as rough endoplasmic reticulum, mitochondria, glycogen granules, and euchromatin in the nucleus (Figure 3(a)). Ultrastructural analysis of the CCl_4_ group revealed chronic cell injury, characterized by the presence of numerous swollen mitochondria and peroxisomes, dilated rER, and several vacuoles including autophagosomes, as well as condensed chromatin with areas of nucleic acid lyse that are characteristic of a necrotic process (Figures 3(b), 3(c), and 3(d)). The hepatocytes of the CCl_4_ + DEC group exhibited typical morphology with well-preserved organelles, euchromatic nuclei, and numerous mitochondria in the cytoplasm and many cisterns of rER, similar to those observed in the control group (Figures 3(e) and 3(f)).

### 3.4. Immunohistochemistry and Immunofluorescence

The release of inflammatory cytokines can activate other hepatic cells (endothelial cells, stellate cells, and hepatocytes) and induce the expression of chemokines, cytokines, and enzymes that attract and activate inflammatory cells from the circulation [35, 36].

**Figure 4:**
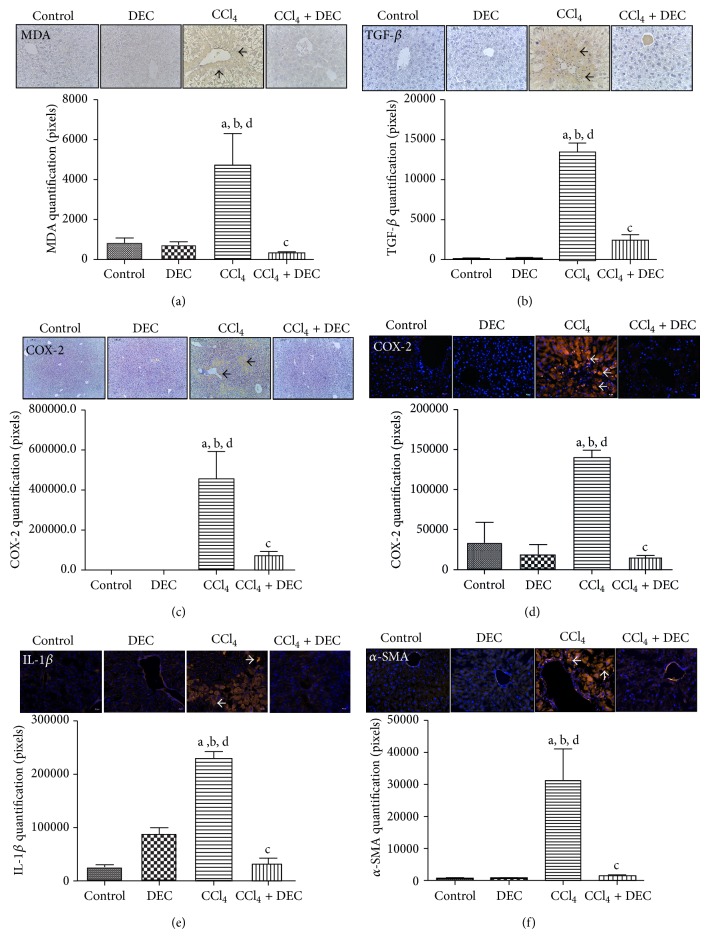
Immunohistochemistry (IHC) and immunofluorescence (IF) for (a) MDA, (b) TGF-*β*, (c) and (d) COX-2, (e) IL-1*β*, and (f) *α*-SMA. Control group, 50 mg/kg DEC-treated group; CCl_4_ group, DEC + CCl_4_ group. Black arrows indicate IHC labeling. White arrows show IF staining. IHC and IF quantification (mean ± S.D., *n* = 5). ^a^ *P* < 0.05 when compared with control group; ^b^ *P* < 0.05 when compared with DEC group; ^c^ *P* < 0.01 when compared with CCl_4_ group; ^d^ *P* < 0.05 when compared with CCl_4_ + DEC group.

The administration of CCl_4_ produced severe liver damage as indicated by a marked increase in malondialdehyde (MDA) (Figure 4(a)). Immunohistochemical staining showed that the MDA in the control and DEC groups was not immunopositive. On the other hand, MDA was highly accumulated in the hepatic tissue, especially in perivenular areas of the CCl_4_ group. In contrast, MDA was depleted in the CCl_4_ + DEC group. Immunostaining quantitation of MDA was performed using GIMP 2.6 image software.

TGF-*β* has been characterized as an important cytokine which mediates hepatic fibrogenesis. Kupffer cells and hepatic stellate cells (HSCs) are the major producers of the extracellular matrix in the fibrotic liver and are fundamental in liver fibrogenesis [23, 37]. TGF-*β* immunohistochemistry (Figure 4(b)) in the control and DEC groups confirmed no immunopositivity for TGF-*β*. However, the CCl_4_ group exhibited a significant increase in TGF-*β*, mainly in fibrotic areas and in the HSCs. A significant reduction of this cytokine was observed in the CCl_4_ + DEC group, suggesting that DEC is involved in the decrease of hepatic fibrosis.

COX-2 expression is increased in inflammatory conditions, which results in the induction of different stimuli, including proinflammatory cytokines such as TNF-*α* and IL-1*β* [34]. COX-2 inhibition has a hepatoprotective effect on liver injuries induced by carbon tetrachloride [38].

COX-2 physiological levels were observed in the control group and the DEC group (Figures 4(c) and 4(d)). However, significantly increased expression levels of this enzyme were found in the CCl_4_ group. COX-2 immunoreactivity was mainly observed in fibrotic areas, with a predominance of pericentral staining. COX-2 expression was significantly reduced in the CCl_4_ + DEC group. Immunostaining quantitation, immunohistochemistry, and immunofluorescence for COX-2 were performed using GIMP 2.6 imaging software and are illustrated in Figures 4(c) and 4(d), respectively.

In the immunofluorescence analysis, the control and DEC groups were not immunopositive to IL-1*β* (Figure 4(e)). However, in the CCl_4_ group, a high expression of the proinflammatory cytokine IL-1*β* was observed. This expression was significantly lower in the CCl_4_ + DEC group than in the CCl_4_ group (Figure 4(e)).

Activated HSCs are transformed into myofibroblasts, producing *α*-SMA and increasing the secretion of collagen fibers, which results in the deposition of fibrotic matrix constituents [39].

In the present study, the activation of HSCs was identified by the increased expression of *α*-SMA in the CCl_4_ group (Figure 4(f)), while the expression of *α*-SMA was significantly reduced in the CCl_4_ + DEC group (Figure 4(f)). This indicates that DEC may reduce this activation by preventing the initiation of the HSC fibrous process. This finding correlates with the Sirius-red staining results (Figure 2).

### 3.5. Expression Analysis (Western Blot) of the Pro- and Anti-Inflammatory Markers IL10, IL-1*β*, NF-*κ*B, COX-2, IFN*γ*, and TGF-*β*

**Figure 5:**
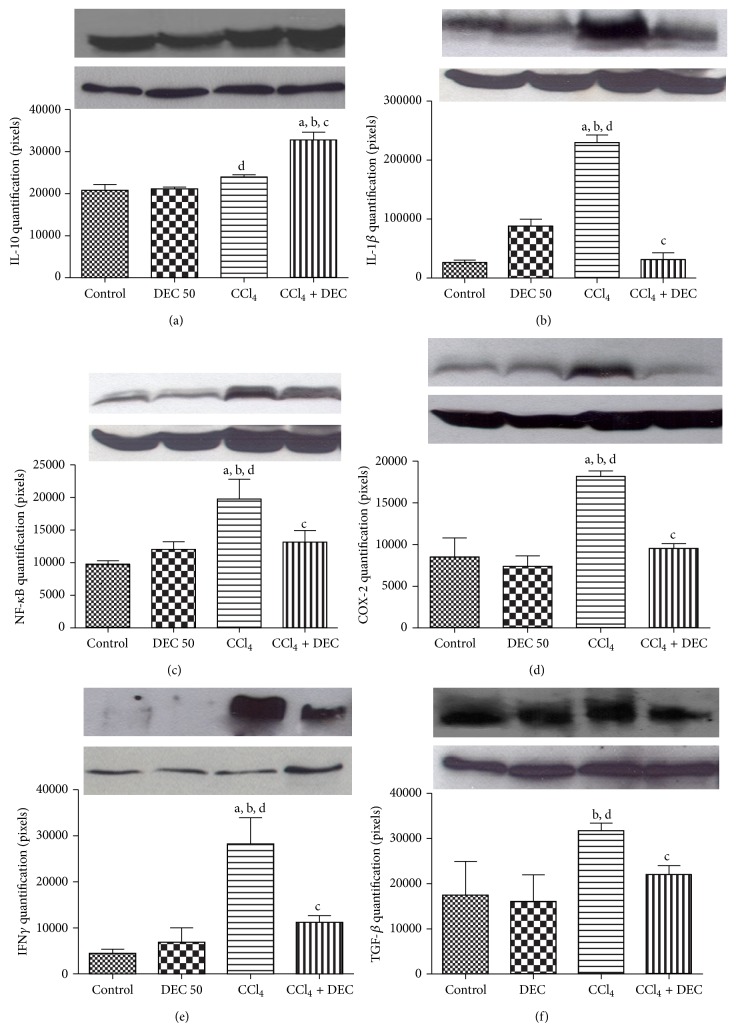
Markers of liver injury. (a) IL-10, (b) IL-1*β*, (c) NF-*κ*B, (d) COX-2, (e) IFN*γ*, and (f) TGF-*β* expressions. Each value represents the mean ± S.D. for 5 animals per group. Data were analyzed using one-way ANOVA followed by Dunnett's test and Tukey's test. ^a^ *P* < 0.05 when compared with control group; ^b^ *P* < 0.01 when compared with DEC group; ^c^ *P* < 0.01 when compared with CCl_4_ group; ^d^ *P* < 0.01 when compared with CCl_4_ + DEC group. The results were confirmed in three sets of experiments.

The ability of IL-10 (18 kDa) to modulate the inflammatory response and limit hepatotoxicity has been demonstrated in several models of liver injury [40]. According to the results of the present study, the CCl_4_ + DEC group showed a significant increase in the expression of anti-inflammatory cytokines, when compared to the other groups (Figure 5(a)).

Interleukin IL-1*β* (17 kDa) showed a significant increase in expression in the CCl_4_ group. In the CCl_4_ + DEC group, the expression of this cytokine was significantly decreased (Figure 5(b)).

Similarly, low expression of p65-NF-*κ*B (60 kDa) was observed in the control and DEC groups. In contrast, significantly elevated levels of expression of NF-*κ*B were observed in the CCl_4_ group, whereas NF-*κ*B was significantly reduced in the CCl_4_ + DEC group, when compared to the inflamed group (Figure 5(c)).

The DEC group did not alter COX-2 (42 kDa) expression when compared to the control group. The levels of COX-2 in the CCl_4_ group increased, when compared to the control and DEC groups. However, the CCl_4_ + DEC group significantly reduced the expression of this enzyme, indicating an anti-inflammatory effect against chronic liver inflammation (Figure 5(d)), thereby confirming the results obtained in the immunohistochemistry and immunofluorescence (Figures 4(c) and 4(d)).

IFN*γ* expression was not observed in the control and DEC groups. However, a significant increase was observed in the CCl_4_ group. The CCl_4_ + DEC group decreased IFN*γ* levels significantly (Figure 5(e)).

TGF-*β* was detected in all groups. An increase was observed in the CCl_4_ group due to the liver injury caused by CCl_4_ (Figure 5(f)). However, a reduction in expression was observed in the CCl_4_ + DEC group, suggesting a beneficial DEC effect against chronic inflammation, as well as the antifibrotic effect, confirming the data obtained in the immunohistochemistry (Figure 4(b)).

### 3.6. Analysis of the mRNA Expression of COX-2 and iNOS

**Figure 6:**
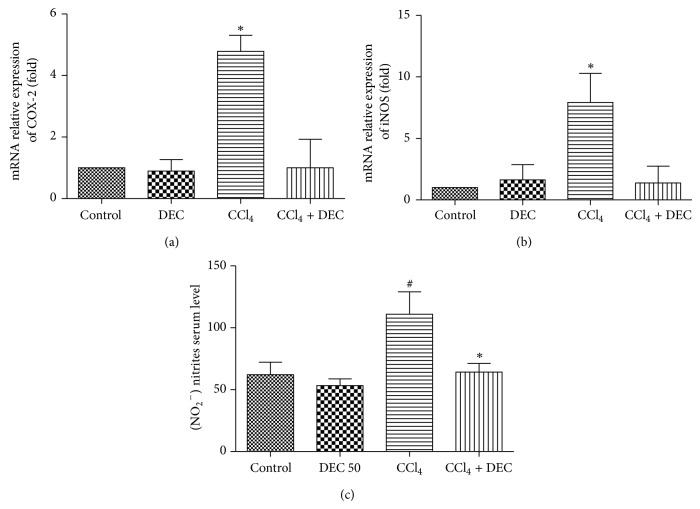
(a) Relative expression of mRNA COX-2; (b) mRNA expression of iNOS. CCl_4_ group significantly increased mRNA COX-2 and iNOS expressions when compared with other groups. An asterisk represents a significant difference of the CCl_4_ group between groups. (c) Assessment of NO in serum through the measure of total nitrite metabolites. Quantification of NO (mean ± S.D., *n* = 6). ^#^ *P* < 0.05 when compared with control group; ^*^ *P* < 0.02 when compared with CCl_4_ group.

The levels of COX-2 and iNOS mRNA expression increased 4.6-fold and 9.7-fold over the CCl_4_ group, respectively, when compared with the control group. COX-2 and iNOS mRNA expression were significantly attenuated in the CCl_4_ + DEC group (Figures 6(a) and 6(b)).

### 3.7. Nitric Oxide Levels

With regard to the level of NO_2_^−^ in the serum, a significant increase was detected in the NO metabolites in the CCl_4_ group (110.9 ± 18.23), when compared to the other groups. In the CCl_4_ + DEC group (64.22 ± 6.96), a significant decrease was observed when compared to the CCl_4_ group (Figure 6(c)).

## 4. Discussion

Chronic inflammation leads to continuous hepatocyte damage and subsequently hepatic fibrosis [23, 36, 41].

The response is generalized, with features common to multiple organ systems. In the liver, a variety of different types of injury lead to inflammation and fibrogenesis, implying a common pathogenesis. The recruitment and migration of Kupffer cells and HSCs are critical events in the development of liver inflammation and fibrosis [32].

Although a number of specific therapies for patients with different liver diseases have been successfully developed, including antiviral therapies for patients with hepatitis B and hepatitis C infections, specific and effective hepatic anti-inflammatory and antifibrotic therapy remains elusive. Elucidation of these mechanisms has been of fundamental importance in highlighting new potential therapies [2].

According to Rocha et al. [15], treatment of alcoholic animals with DEC 50 mg/kg prevented lipid accumulation and promoted a reduction of the inflammatory infiltrates and necrosis areas caused by alcoholism. In addition, DEC at 25 and 50 mg/kg was used to treat malnourished animals and prevented lipid accumulation, while promoting a reduction in the damage caused by malnutrition [30].

A CCl_4_-induced hepatic injury is a widely used experimental model for anti-inflammatory and hepatoprotective drug screening, promoting hepatic pathology similar to that observed in humans [42–45]. The CCl_4_ exerts effects on immunity cells such as T-cells, NK cells, macrophages, phagocytes, and lymphatic organs, besides increasing cytokines inflammatory (IL-2, TGF-beta) [46, 47]. The present paper used the CCl_4_-injury hepatic model to analyze the DEC therapeutic potential in preventing chronic hepatotoxicity by attenuating the inflammatory response and fibrosis in the liver.

Gonzàlez et al. [48] showed that rats with liver inflammation induced by CCl_4_ had less liver damage after DEC treatment at 25 and 50 mg/kg. The animals had well-preserved organelles and a membrane system of hepatocytes, showing that DEC had a protective effect. In the present study, the CCl_4_ group (CCl_4_) exhibited an evident inflammatory process, as well as necrosis and fibrosis. However, a reduction of inflammatory infiltrates, liver necrosis, and fibrosis was observed in the CCl_4_ + DEC group. Therefore, DEC treatment caused an evident reduction of hepatic damage, corroborating the results of previous studies.

Several studies have shown that chronic liver inflammation and fibrosis is related to the chronic phase of Kupffer cell accumulation, HSC activation, and collagen deposition [49–51]. The present study demonstrated that there was a significant increase in collagen fiber deposition in liver tissue in the CCl_4_ group. It has been shown that hepatic fibrosis is largely the result of disorder in the homeostasis of the synthesis, deposition, degeneration, and absorption of collagens. In hepatic fibrosis, myofibroblasts characteristically assume the ability to remodel the extracellular matrix (ECM) via the production of ECM proteins in the liver [52, 53]. After DEC treatment, a significant decrease of fiber collagen depositions was assessed by Sirius-red staining. Therefore, the present study suggests that DEC has an antifibrotic effect.

Nitric oxide (NO) is known to react with superoxide radical, forming peroxynitrite, an even more potent oxidizing agent [54, 55]. Peroxynitrite can react directly with sulfhydryl residues in cell membranes and DNA, leading to lipid peroxidation and cytotoxicity [54, 56]. The present study illustrated that there was an elevation in serum nitric oxide levels in CCl_4_-treated mice and that DEC treatment was capable of attenuated serum NO levels. This may be related to the reduction of iNOS levels in hepatic tissue.

The iNOS enzyme is expressed in hepatocytes and inflammatory cells during the development of acute and chronic diseases [57, 58] but the role of NO^•^ in tissue damage is still controversial. Although a hepatoprotective function of iNOS has been observed in different types of liver injury, including chronic CCl_4_-intoxication [59, 60], hepatotoxic effects of iNOS have also been reported [61]. The iNOS gene is expressed by hepatocytes in a number of physiological and pathophysiological conditions affecting the liver, including septic, hemorrhagic shock and alcoholism [62]. It seems that iNOS-derived NO^•^ could contribute to nitrosative stress [63] but also regulate proinflammatory gene expression, thereby contributing to inflammatory liver injuries [45]. Rocha et al. [15] demonstrated that DEC suppresses the activation of NF-*κ*B and downstream proinflammatory mediators, including iNOS. The results of the present study suggest antinitrosative and anti-inflammatory effects of DEC in CCl_4_-induced liver injuries.

It is known that different types of cells and cytokines involved in inflammation participate in chronic hepatic injuries. These cytokines, such as extracellular stimulants, regulate the genetic expression of various factors through interactions with transcription-modulating factors. NF-*κ*B has been considered a modulating factor, which regulates immunologic reactions and apoptosis, mediating acute and chronic inflammatory reactions [64]. NF-*κ*B reportedly plays an important role in chronic liver injuries resulting from the action of CCl_4_ [65]. The main action of NF-*κ*B in liver injuries is to mediate the release of cytotoxic cytokines and inflammatory cytotoxins. The present study provides additional findings supporting the activity of DEC in modulating the severity of inflammation through several key transcription factors such as NF-*κ*B.

Several studies have demonstrated that certain nonsteroidal anti-inflammatory drugs (NSAIDs), such as sodium salicylate, sulindac, ibuprofen, and flurbiprofen, exhibit anti-inflammatory effects independent of the cyclooxygenase pathways. Much interest has focused on NF-*κ*B as a potential target for certain NSAIDS [66–68]. Similarly, DEC also exhibits anti-inflammatory effects while decreasing NF-*κ*B expression.

Over one hundred target genes for NF-*κ*B have been identified during the past few years. One of them is the COX-2 gene. It is known that COX-2 is responsible for inflammatory reactions and carcinogenesis of hepatocellular carcinoma [69]. COX is another key molecule in the inflammatory pathway and it is induced by several stimuli, including cytokines and mitogens. COX-2 expression results in the release of prostaglandin at the site of inflammation. In the present study, the levels of COX-2 protein and mRNA expression increased in the CCl_4_ group and DEC markedly attenuated these increases. High levels of iNOS and COX-2 cause the production of high concentrations of NO and eicosanoids, through the initiation of the COX-prostanoid pathway [26, 70], which leads to cellular inflammation, necrosis, and fibrosis. Taken together, the results of the present study suggest that DEC largely regulates the production of iNOS and COX-2 on a transcriptional level.

Although the results of liver injuries are associated with the CCl_4_ metabolism, secondary damage occurs due to the inflammatory process initiated by Kupffer cell activation [9, 71]. Kupffer cells activate the release of inflammatory mediators, including TNF-*α* and IL-1*β*. Interleukin-1*β* is a potent inflammatory cytokine that is involved in the synthesis of prostaglandins, macrophage activation, and the induction of neutrophil infiltration, as well as several aspects of inflammation [13, 72]. The present study showed that DEC treatment effectively reduced CCl_4_-induced liver injuries through the inhibition of IL-1*β*.

The results of the present study demonstrated that there was a significant elevation in MDA content in the liver tissue of CCl_4_-treated mice. This enhanced lipid peroxidation led to tissue damage and the failure of antioxidant defense mechanisms. In the present study, there was a significant elevation in liver MDA content in the CCl_4_ group and a significant reduction in the group treated with DEC, which suggests enhanced lipid peroxidation leading to tissue damage and the failure of antioxidant defense mechanisms to prevent the formation of excessive free radicals [73]. Therefore, since DEC treatment reduced MDA levels, it probably exhibits an antioxidant action.

IFN*γ* plays a crucial role in modulating immune responses. Previous studies have reported that the level of IFN*γ* was positively correlated with serologic markers of hepatic injury and fibrogenesis [74]. The reason for elevated levels of IFN*γ* in the development of hepatic injuries and fibrogenesis remains unclear [75]. The present study showed that IFN*γ* expression decreased after DEC treatment, suggesting an advantageous response against the CCl_4_-caused injury in the animals.

Certain immunomodulatory cytokines can contribute to the establishment of an anti-inflammatory state in the liver and ameliorate injuries. One such example is IL-10, which exhibits potent anti-inflammatory and immunosuppressive properties, decreasing the production of proinflammatory cytokines, including TNF-*α* and IL-1*β* [76]. The ability of IL-10 to modulate the inflammatory response and to limit hepatotoxicity has been shown in several models of liver injury [77–79]. IL-10 may also have antifibrogenic properties linked to the downregulation of profibrogenic cytokines, such as TGF-*β* [80]. Increased endogenous IL-10 expression ameliorates chronic inflammatory burst and subsequent liver fibrosis after repeated stimulation with CCl_4_ [40]. Similarly, the results of the present study showed that DEC increased IL-10 expression during CCl_4_ intoxication and decreased inflammation and the subsequent fibrogenic response in the liver.

Chronic damage, such as a liver inflammatory response, results in fibrosis in conjunction with the accumulation of ECM proteins, characteristic of most types of chronic liver disease [81, 82]. HSCs are the primary ECM-producing cells in an injured liver [83]. HSCs that are activated or transdifferentiated into myofibroblast-like cells acquire contractile, proinflammatory, and fibrogenic properties [49, 84, 85]. Key cytokines are involved in liver fibrosis, regulating the inflammatory response to injury and modulating hepatic fibrogenesis. TGF-*β* and *α*SMA appear to be crucial mediators in human fibrogenesis [86]. The stimulation of activated HSCs by TGF-*β* is believed to be a crucial fibrogenic response in liver fibrosis for the following reasons: higher TGF-*β* expression in activated HSC; potency of TGF-*β* to upregulate ECM expression; higher expression of TGF-*β* receptors in relation to HSCs; and increased expression of TIMP-1/TGF-*β* liver fibrosis induced [87, 88]. Strategies aimed at disrupting TGF-*β* synthesis and/or reducing the signaling pathways decrease fibrosis in experimental models [89]. The immunohistochemistry and immunofluorescence analysis showed that TGF-*β* increased significantly in the CCl_4_ group compared to the others groups, in contrast with CCl_4_ + DEC which significantly reduced TGF-*β* expression, confirming previous observations.

Alpha smooth muscle actin (*α*-SMA) is a protein that participates in the processes of tissue repair, present at a particular stage of fibroblast differentiation. These cells were shown to be responsible for the contraction of granulation tissue and fibrotic lesions [90]. Activated HSCs are responsible for liver damage, producing type I collagen in hepatic fibrosis and also expressing *α*-SMA filaments. The activation of HSCs is an essential characteristic of hepatic fibrosis and this activation is indicated by the expression of *α*-SMA, although it can be expressed in other cell types [91]. A significantly higher labeling for *α*-SMA was observed in the CCl_4_ group than in the control group. On the other hand, low levels of *α*-SMA were observed in the CCl_4_ + DEC group. These results indicate that DEC can inhibit TGF-*β* and *α*-SMA expression, consequently reducing the impact of the proliferation and activation of HSCs. These results are in agreement with previous observations that DEC treatment significantly reduced fiber collagen depositions, confirming that DEC has an antifibrotic effect.

In summary, the present study demonstrated for the first time that DEC can protect against CCl_4_-induced chronic hepatotoxicity and exerts an antifibrotic effect through reduction of inflammatory markers as TGF-*β*, *α*-SMA, and Sirius-red staining, revealing a clinical potential of DEC for therapeutic antifibrotic applications.
